# Clozapine metabolites protect dopaminergic neurons through inhibition of microglial NADPH oxidase

**DOI:** 10.1186/s12974-016-0573-z

**Published:** 2016-05-16

**Authors:** Lulu Jiang, Xuefei Wu, Shuo Wang, Shih-Heng Chen, Hui Zhou, Belinda Wilson, Chun-Yang Jin, Ru-Band Lu, Keqin Xie, Qingshan Wang, Jau-Shyong Hong

**Affiliations:** Neuropharmacology Section, Laboratory of Neurobiology, National Institute of Environmental Health Sciences, National Institutes of Health, Research Triangle Park, NC 27709 USA; Institute of Toxicology, School of Public Health, Shandong University, Jinan, Shandong 250012 China; Department of Physiology, Dalian Medical University, Dalian, Liaoning 116044 China; Department of Occupational and Environmental Health, School of Public Health, Peking University, Beijing, 100191 China; Center for Drug Discovery, Research Triangle Institute, Research Triangle Park, NC 27709 USA; Department of Psychiatry, Institute of Behavioral Medicine, Institute of Allied Health Sciences and Addiction Research Center, National Cheng Kung University Hospital, College of Medicine, National Cheng Kung University, Tainan, 70101 Taiwan; Center for Neuropsychiatric Research, National Health Research Institutes, Miaoli, 35035 Taiwan; Department of Occupational and Environmental Health, School of Public Health, Dalian Medical University, Dalian, Liaoning 116044 China

**Keywords:** Neuroinflammation, NADPH oxidase, Clozapine metabolites, Parkinson’s disease, Neuroprotection

## Abstract

**Background:**

Clozapine, an atypical antipsychotic medication, has been effectively used to treat refractory schizophrenia. However, the clinical usage of clozapine is limited due to a high incidence of neutropenia or agranulocytosis. We previously reported that clozapine protected dopaminergic neurons through inhibition of microglial activation. The purpose of this study was to explore the neuroprotective effects of clozapine metabolites clozapine *N*-oxide (CNO) and *N*-desmethylclozapine (NDC), as well as their propensity to cause neutropenia.

**Methods:**

The primary midbrain neuron-glia culture was applied to detect the neuroprotective and anti-inflammatory effect of clozapine and its metabolites in lipopolysaccharide (LPS) and MPP^+^-induced toxicity. And the subsequent mechanism was demonstrated by gp91^*phox*^ mutant cell cultures as well as microgliosis cell lines. In vivo, to confirm the neuroprotective effect of clozapine and CNO, we measured the dopaminergic neuronal loss and rotarod motor deficits in 1-methyl-4-phenyl-1,2,3,6-tetrahydropyridine (MPTP)-generated mouse Parkinson’s disease (PD) model. The neutropenia or agranulocytosis of clozapine and its metabolites was illustrated by white blood cell count of the treated mice.

**Results:**

We found that, in midbrain neuron-glia cultures, CNO and NDC were more potent than clozapine in protecting dopaminergic neurons against LPS and MPP^+^-induced toxicity. CNO and NDC-afforded neuroprotection was linked to inhibition of microglia-mediated neuroinflammation, as demonstrated by abolished neuroprotection in microglia-depleted cultures and their capacity of inhibiting LPS-induced release of proinflammatory factors from activated microglia. NADPH oxidase (NOX2) was subsequently recognized as the main target of CNO and NDC since genetic ablation of gp91^*phox*^, the catalytic subunit of NOX2, abolished their neuroprotective effects. CNO and NDC inhibited NOX2 activation through interfering with the membrane translocation of the NOX2 cytosolic subunit, p47^*phox*^. The neuroprotective effects of CNO were further verified in vivo as shown by attenuation of dopaminergic neurodegeneration, motor deficits, and reactive microgliosis in MPTP-generated mouse PD model. More importantly, unlike clozapine, CNO did not lower the white blood cell count.

**Conclusions:**

Altogether, our results show that clozapine metabolites elicited neuroprotection through inactivation of microglia by inhibiting NOX2. The robust neuroprotective effects and lack of neutropenia suggest that clozapine metabolites may be promising candidates for potential therapy for neurodegenerative diseases.

**Electronic supplementary material:**

The online version of this article (doi:10.1186/s12974-016-0573-z) contains supplementary material, which is available to authorized users.

## Background

Clozapine, an atypical antipsychotic medication, is widely used for treatment-resistant schizophrenia patients, demonstrating greater efficacy against both positive and negative symptoms, compared with typical antipsychotics [1]. Other benefits of clozapine application include the lack of extrapyramidal adverse effects [2] and the ability to improve cognitive function [3]. Despite these advantages, the usage of clozapine is rather restricted due to a relatively high risk of neutropenia or agranulocytosis [4–6]. Currently, the mechanism responsible for neutrophil toxicity of clozapine is not fully understood. *N*-Desmethylclozapine (NDC) and clozapine *N*-oxide (CNO), as well as reactive nitrenium intermediate, are the known major metabolites of clozapine in humans [7, 8]. In vitro studies demonstrated that reactive nitrenium intermediates but not clozapine itself or NDC/CNO is toxic to neutrophils [9, 10]. In addition, it is generally accepted that NDC and CNO are different in terms of receptor activity as CNO has been proved to be pharmacologically inert [11, 12] to the receptors for which clozapine and NDC function as agonists or antagonists [11, 13, 14].

Microglia, the resident innate immune cells in the central nervous system (CNS), serve immune surveillance function in physiological conditions. In response to certain cues, such as brain injury or immunological stimuli, microglia are readily activated and play a central role in the process called “neuroinflammation.” It is now widely accepted that dysregulated neuroinflammation featured by microglial over-activation has significant impacts on the pathogenesis of neurodegenerative disorders such as Parkinson’s disease (PD) [15, 16] and Alzheimer’s disease (AD) [17–21]. Furthermore, increasing evidence also suggests an association of neuroinflammation with several psychiatric disorders, including schizophrenia (SCZ), autism, depression, and anxiety disorders [22–24].

We have previously reported that clozapine protects dopaminergic neurons from inflammation-induced damage by inhibiting microglial NADPH oxidase (NOX2, a superoxide-producing enzyme) in primary cell cultures [25]. The main purpose of this study was to determine whether the two different metabolites could be less toxic than clozapine and preserve the same neuroprotective and anti-inflammatory effects of the parent drug. In this study, we found that both CNO and NDC protected DA neurons through suppression of microglia-mediated neuroinflammation both in vitro and in vivo, where inactivation of microglial NOX2 played a central role. More importantly, unlike clozapine, CNO displayed no effects on blood neutrophils. These effects revealed a novel bioactivity of CNO and NDC without the propensity of producing neutropenia.

## Methods

### Animals

Time-pregnant Fisher F344 rats were provided by the Charles River Laboratories (Raleigh, NC). Wild-type C57BL/6J (gp91^*phox*+/+^) and phagocytic NADPH oxidase (NOX2)-deficient (gp91^*phox*−/−^) mice were obtained from the Jackson Laboratory (Bar Harbor, ME). Breeding of time-pregnant mice was performed with accuracy of 0.5 day. All the mice were euthanized at the desired time points. Housing, breeding, and experimental use of the animals were performed in strict accordance with the National Institutes of Health guidelines. All procedures were approved by the NIEHS animal care and use committee.

### Reagents

N-Desmethylclozapine, 1-methyl-4-phenyl-1,2,3,6-tetrahydropyridine (MPTP), MPP^+^, and Leu methyl ester (LME) were purchased from Sigma-Aldrich (St. Louis, MO). Clozapine and clozapine *N*-oxide were obtained from the NIMH Chemical Synthesis and Drug Supply Program (Research Triangle Institute, RTP NC). Lipopolysaccharide (LPS strain O111:B4) was purchased from Calbiochem (San Diego, CA). WST-1 was purchased from Dojindo Laboratories (Gaithersburg, MD). Cell culture ingredients were obtained from Invitrogen (Carlsbad, CA). [^3^H]DA was purchased from PerkinElmer Life Sciences (Boston, MA). The polyclonal anti-tyrosine hydroxylase (TH) antibody was purchased from CHEMICON International (Temecula, CA). The polyclonal ionized calcium-binding adaptor molecule 1 (Iba-1) antibody was purchased from Wako Chemicals USA (Richmond, VA). The monoclonal CD11b antibody was purchased from AbDSerotec (Raleigh, NC). The biotinylated secondary antibodies were purchased from Vector Laboratories (Burlingame, CA).

### Animal treatment

Eight-week-old male C57BL/6J mice received daily MPTP injections (20 mg/kg, s.c.) for six consecutive days. One day prior to MPTP injection, clozapine or CNO (1 mg/kg, s.c.) was administered twice daily for 21 consecutive days. Eight days after initial MPTP injection, five mice from each group were sacrificed for the detection of microglial activation in substantia nigra (SN). Fourteen days after initial MPTP injection, the protective effects of clozapine and CNO against MPTP-induced motor deficits were measured by the accelerating rotarod test. At 8 and 21 days after the first injection of MPTP, respectively, mice were euthanized, and brains were removed and postfixed in 4 % paraformaldehyde overnight at 4 °C. Brains were then placed into 30 % sucrose/PBS solution at 4 °C until the brains sank to the bottom of the container. Coronal sections including SN pars compacta (SNpc) were cut on a −20 °C frozen sliding microtome (Thermo Scientific, microm HM525) into 40-μm transverse free-floating sections.

### Blood analysis

Twenty-one days after the first injection of MPTP, the mice were euthanized and eyeball blood was collected in a 1.5-ml heparin tube. After a fully vortex, the number of different types of cells in the blood was counted by an Automatic Blood cell Counter (Model: CA-800, SANKYO Inc.).

### Rotarod test

The rotarod behavior was measured as described previously using a Rota-Rod (ZS-RDM R03-1, Zhong-Shi Inc., Beijing, China). The parameters of the rotarod system were set as accelerating speed from 4 to 40 rpm in 300 s [26, 27]. Mice received three consecutive trials. The rest period between each trial was 30 min. The mean latency for the last two trails was used for the analysis.

### Immunostaining

The free-floating brain sections or fixed cells in 24-well culture plate were immune-blocked with 4–10 % goat serum and then incubated with polyclonal rabbit anti-TH antibody (1:5000 dilution), or Iba-1 antibody (1:5000 dilution), or rat anti-CD11b antibody (1:800 dilution) for 24 h at 4 °C, respectively. Antibody binding was visualized using a Vectastain ABC Kit (Vector Laboratories, Inc) and diaminobenzidine (or with cobalt) tablet as substrate.

Images were recorded with a CCD camera and the MetaMorph software (Molecular Devices). TH immunostaining-reactive (THir) neuron or Iba-1ir microglia number were counted according to published protocol [28] and was carried out by at least two investigators without knowledge of the treatment. For immunocytochemistry staining in 24-well cell culture, three to six wells per treatment condition were used, and results from three to five independent experiments were obtained.

### Primary cell cultures

Primary neuron-glia cultures were prepared as described previously [29]. In brief, dissociated cells from the ventral mesencephalon of embryonic day 14 ± 0.5 Fischer 334 rats, gp91^*phox+/+*^or gp91^*phox−/−*^ mice were seeded at 5.5 × 10^5^ cells/well (rat) or 6.5 × 10^5^ cells/well (mice) in poly-d-lysine-coated 24-well plates, respectively. The cultures were maintained at 37 °C in the incubator with 5 % CO_2_ and 95 % air in minimum essential medium. The cultures were ready for experiments 7 days later, when the cultures became mature and stable of each cell component (astrocytes ~50 %, neurons ~40 %, and microglia ~10 %) as described previously [29, 30]. Microglia-depleted neuron-glia cultures were obtained by depleting microglia in neuron-glia cultures with 1.5 mM of LME 48 h after seeding (~45 % neurons and ~55 % astrocyte), as described previously [31].

Mixed-glia cultures were prepared from whole brains of postnatal day 1 rats as reported before [32]. Briefly, disassociated cells were seeded into 24-well (1 × 10^5^/well) or 96-well (5 × 10^4^/well) culture plates and maintained in 1 ml/well or 0.2 ml/well of Dulbecco’s modified Eagle’s medium (DMEM)/F-12 medium. The medium was changed every 3 days. On 11–12 days after plating, the cultures were mature and stable with different cell components [29, 30] (astrocytes ~80 %, GFAP immunopositive cells; microglia ~20 % OX-42 immunopositive cells) ready for drug treatment or superoxide assay.

### Culture treatment

Rat mesencephalic neuron-glia cultures or microglia-depleted neuron-glia cultures were maintained in the maintenance medium (10 % fetal bovine serum, 10 % horse serum, 0.1 % d-glucose, 1 % none essential AA, Na pyruvate 1 %, l-glutamine 1 %, Pen/Strep1% in MEM) for 7 days untile the cultures became mature (astrocytes ~50 %, neurons ~40 %, and microglia ~10 %). Then, the cultures were pretreated with vehicle or indicated concentrations of CNO, NDC, or clozapine prepared in the serum-reduced treatment medium (2 % fetal bovine serum, 2 % horse serum, Na pyruvate 1 %, l-glutamine 1 %, Pen/Strep1% in MEM) for 30 min before the addition of LPS (15 ng/ml) or MPTP (0.25 μM), which also prepared in the serum-reduced treatment medium. At indicated time points after treatment, the culture supernatant was collected for the detection of inflammatory factors. And 7 days after LPS or MPTP treatment, the protective effects of CNO, NDC, or clozapine against inflammation-elicited damage of DA neurons were determined by quantifying functional changes of [3H]DA uptake capacity and counts of THir neurons.

### Cell lines

The rat microglia HAPI cell line was a gift from Dr. J. R. Connor (Pennsylvania State University, Hershey, PA) [33] and maintained as described previously [34]. Briefly, HAPI cell line were maintained at 37 °C in DMEM (Sigma) supplemented with 10 % fetal bovine serum, 50 U/ml penicillin, and 50 μg/ml streptomycin in a humidified incubator with 5 % CO_2_ and 95 % air. The cells were split or harvested every 3−5 days.

### [^3^H]DA uptake assay

[^3^H]DA uptake assays were performed as described previously [35]. Briefly, cells were incubated for 21 min at 37 °C with 1 μM [^3^H]DA (PerkinElmer Life Sciences) in Krebs-Ringer buffer. Cells were washed with ice-cold Krebs-Ringer buffer three times and then were collected in 1 N NaOH. Radioactivity was determined by liquid scintillation counting. Nonspecific DA uptake observed in the presence of mazindol (10 μM) was subtracted.

### NO and TNF-α assays

The production of nitric oxide (NO) was determined by measuring accumulated levels of nitrite in the supernatant with Griess reagent, and the release of tumor necrosis factor-α (TNF-α) was measured with a TNF-α ELISA kit from R&D Systems (Minneapolis, MN) following manufacture’s protocol.

### Measurement of superoxide

The production of superoxide was assessed by measuring the SOD-inhibitable reduction of the tetrazolium salt WST-1 [36]. Briefly, primary neuron-glia cultures were washed twice with HBSS balanced salt solution and then pretreated with indicated concentrations of clozapine metabolites dissolved in HBSS for 15 min. Immediately after the addition of LPS, 50 μl of HBSS with or without SOD (50 U/ml) was added to each well along with 50 μl of WST-1 (1 mM) in HBSS. The absorbance at 450 nm was read with a SpectraMax Plus microplate spectrophotometer (Molecular Devices Sunnyvale). The amount of SOD-inhibitable superoxide was calculated and expressed as percentage of vehicle-treated control cultures.

### Cell extracts

Whole cell lyses from HAPI microglia were prepared with lysis buffer (Cell Signaling, Danvers, MA). Subcellular fractionation was performed as described previously [37]. For subcellular fractions, HAPI microglia were lysed in hypotonic lysis buffer (1 mM Tris, 1 mM KCl, 1 mM EGTA, 1 mM EDTA, 0.1 mM DTT, 1 mM PMSF, and 10 μg/ml cocktail protease inhibitor) and then subjected to Dounce homogenization (20–25 St, tight pestle A). The lysates were centrifuged at 1600×*g* for 15 min; the supernatant was centrifuged at 100,000×*g* for 30 min. The pellets solubilized in 1 % nonidet P-40 hypotonic lysis buffer were used as membranous fraction.

### Western blot analysis

For western blot analysis, equal amounts of protein were separated by 4−12 % Bis-Tris Nu-PAGE gel and transferred to polyvinylidenedifluoride membranes. The membranes were blocked with 5 % nonfat milk and incubated with rabbit antibodies (1:1000) against Iba-1, p47^*phox*^, gp91^*phox*^ (BD Transduction Laboratories), GAPDH, or rat CD11b antibody overnight at 4 °C. The next day, membranes were incubated with HRP-linked secondary anti-rabbit or rat IgG (1:3000) for 2 h at room temperature. ECL reagents (Amersham Biosciences) were used as a detection system.

### Statistical analysis

All group data are expressed as mean ± standard error of mean (SEM). Group means were compared using one-way analysis of variance (ANOVA) with treatment as the independent variable. When ANOVA showed a significant difference, pairwise comparisons between group means were examined by Dunnett’s post hoc test. Differences were considered significant at *p* value <0.05. Statistical analysis was performed using GraphPad Prism version 6.00 for Windows.

## Results

### CNO and NDC protect DA neurons from LPS-induced neurotoxicity

**Fig. 1:**
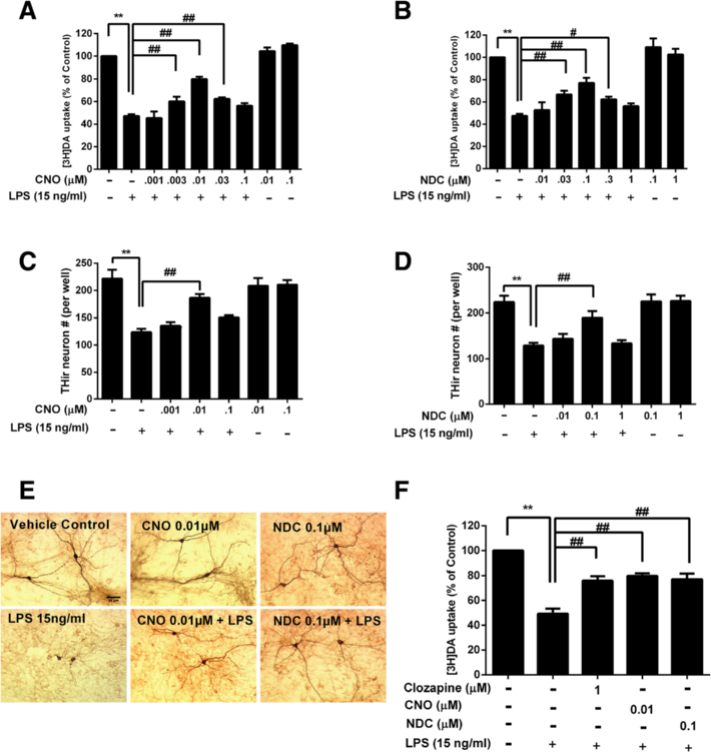
Potent effects of CNO and NDC in protecting DA neurons against LPS-induced neurotoxicity. Rat mesencephalic neuron-glia cultures were pretreated with vehicle or indicated concentrations of CNO, NDC, or clozapine for 30 min before the addition of LPS (15 ng/ml). Seven days after LPS treatment, the protective effects of CNO, NDC, or clozapine against inflammation-elicited damage of DA neurons were determined by quantifying functional changes of [^3^H]DA uptake capacity (**a**, **b**, and **f**) and counts of THir neurons (**c**, **d**). **E** Representative images of anti-TH immunostaining in cultures were shown. Results are expressed as the mean ± SEM or as a percentage of vehicle controls (mean ± SEM) from five independent experiments performed in triplicate. **p < 0.01, ^#^ *p* < 0.05, ^##^ *p* < 0.01. *Scale bar* = 50 μm

To investigate whether clozapine metabolites CNO and NDC display neuroprotective effects, we used inflammation-elicited neurotoxicity in vitro model by treating neuron-glia cultures with *Escherichia coli* endotoxin LPS. Both dopamine uptake capacity and cell counts of THir neurons were performed to determine the function and survival of DA neurons 7 days after LPS treatment. Results showed that pretreatment with CNO or NDC protected DA neurons against LPS-induced reduction of uptake capacity in a bell-shaped curve (Fig. 1a, b). Similar patterns of dopaminergic neuroprotection by both metabolites were observed in cell number counts and morphological observation of neurites of THir neurons (Fig. 1c–e). CNO and NDC were most effective at the concentration of 0.01 and 0.1 μM, respectively, in restoring DA uptake capacity (from 47.4 % of LPS alone back to 79.9 and 77.1 % by CNO and NDC, respectively) and ameliorating loss of THir neurons (from 57.5 % of LPS alone back to 85.2 and 84.2 % by CNO and NDC, respectively, Fig. 1a–e). However, higher concentrations of two compounds showed lower protective efficacy. It is unlikely due to their direct toxicity on neurons, since both NDC and CNO alone at these doses caused no reduction in either DA uptake function or THir cell number (Fig. 1a–d). Thus, for the rest of studies, the most effective concentrations of CNO (0.01 μM) and NDC (0.1 μM) were used.

To compare the neuroprotective potency of clozapine and its two metabolites, we performed DA uptake assay using the most effective concentrations of each compound: clozapine (1 μM) [25], NDC (0.1 μM), and CNO (0.01 μM). The results showed that NDC and CNO in these concentrations exerted equivalent neuroprotection as clozapine (Fig. 1f).

### Microglia are essential in CNO- and NDC-elicited neuroprotection

**Fig. 2:**
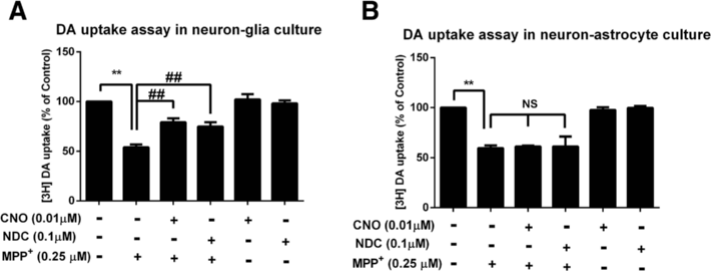
Microglia are required for the neuroprotective effects of CNO and NDC. **a** Rat mesencephalic neuron-glia culture containing 40 % neurons, 50 % astroglia, and 10 % microglia. **b** Microglia-depleted neuron-astrocyte culture containing neurons (~45 %), astroglia (~55 %), and less than 0.01 % microglia were treated with CNO (0.01 μM), NDC (0.1 μM), or vehicle for 30 min prior to the addition of MPP^+^ (0.25 μM). Seven days later, [^3^H]DA uptake assays were performed. Results are expressed as a percentage of the vehicle controls (mean ± SEM) from three independent experiments performed in triplicate. **p < 0.01, ^##^ *p* < 0.01. *NS* not significant

**Fig. 3:**
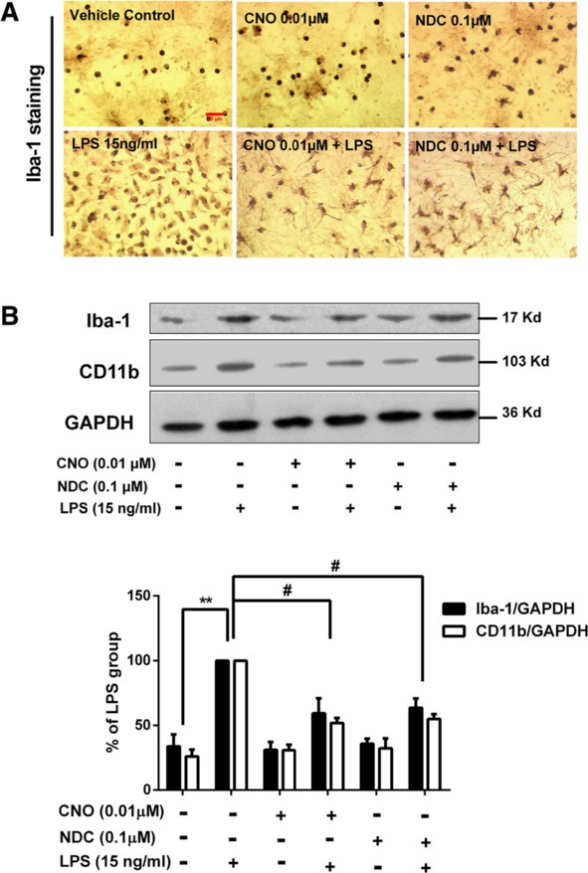
CNO and NDC inhibit LPS-induced microglial activation in vitro. **a** Rats mesencephalic neuron-glia cultures were treated with CNO (0.01 μM), NDC (0.1 μM), or vehicle for 30 min prior to LPS (15 ng/ml) stimulation. Cultures were fixed at 7 days after treatments. Activation of microglia was visualized by immunostaining with anti-Iba-1 antibody and the representative images were shown. **b** The inhibitory effects of CNO and NDC on LPS-induced microglial activation were quantified by assessing the expressions of Iba-1 and CD11b using Western blot. GAPDH was used as loading control. Results are presented as the mean ± SEM from three independent experiments. *p < 0.05. *Scale bar* = 50 μm

**Fig. 4:**
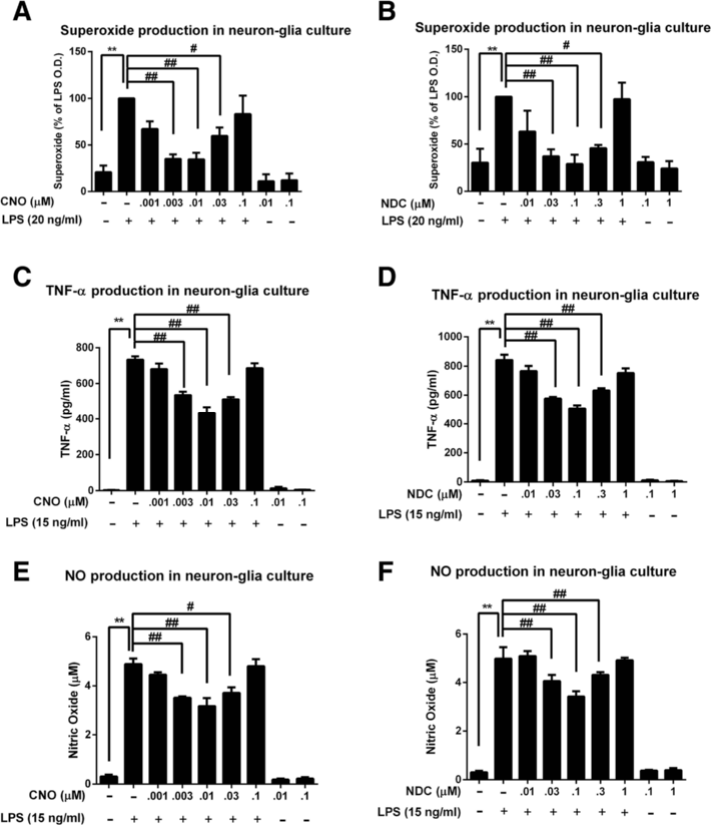
CNO and NDC inhibit LPS-induced release of proinflammatory factors. Rat primary mesencephalic neuron-glia cultures were pretreated with NDC or CNO for 30 min before LPS stimulation. **a**, **b** The production of superoxide was measured by SOD-inhibitable reduction of WST-1. **c**–**f** The levels of TNF-α (**c**, **d**) and NO (**e**, **f**) in the supernatant were measured 3 and 24 h after LPS treatment, respectively. Results are expressed as the mean ± SEM or as a percentage of LPS group (mean ± SEM) from five independent experiments performed in triplicate. **p < 0.01, ^#^ *p* < 0.05, ^##^ *p* < 0.01

To investigate cellular and molecular mechanisms underlying the neuroprotective effects of CNO and NDC, we first determined whether microglia were required. In this study, we compared the neurotoxic effect of 1-methyl-4-phenylpyridinium (MPP^**+**^, the active metabolite of MPTP) in neuron-glia culture with and without microglia (microglia-depleted neuron-astrocyte culture). The reason we used MPP^**+**^, instead of LPS, was because LPS failed to produce dopamine neuron toxicity in microglia-depleted cultures [38, 39]. In this model, DA neurotoxicity caused by both direct MPP^+^ insult on neurons and reactive microglial activation in response to MPP^**+**^-induced neuronal damage [39, 40]. CNO or NDC was shown to afford their neuroprotective effects in neuron-glia culture, but not in microglia-depleted neuron-astrocyte culture from MPP^**+**^-mediated neurotoxicity (Fig. 2). The partial neuroprotective effect of CNO and NDC in MPP^+^-elicited neuron-glia cultures was not observed in microglia-depleted cultures. This result indicated that the presence of microglia in the cultures is essential to show the neuroprotection of CNO and NDC in MPP^+^-treated neuron-glia cultures. The subsequent studies (Figs. 3 and 4) further demonstrated that inhibition of microglial activation and release of proinflammatory immune factors is crucial to the anti-inflammatory and neuroprotective effect of CNO and NDC.

### CNO and NDC inhibit LPS-induced microglial activation

We have further investigated whether CNO and NDC inhibit LPS-induced microglial activation. First, we examined the morphology changes of activated microglia by immunostaining of Iba-1, a specific microglial marker whose expression will be up-regulated during microglial activation [41]. Results showed that LPS stimulation resulted in increased cell number and size of Iba-1-positive microglia and CNO or NDC pretreatment mitigated these changes (Fig. 3a). CNO and NDC treatment alone had no effect on microglial morphology. Western blot analysis of Iba1 and CD11b (the α-chain of β2-integrin receptor, another marker for activated microglia [42]) was then performed to provide the quantitative estimation of microglial activation. Pretreatment of CNO or NDC effectively inhibited the increased expression of Iba-1 and CD11b induced by LPS treatment (Fig. 3b). To exclude the decreases of expression levels of Iba-1 and CD11b which are due to the result of cell toxicity of clozapine metabolites, we further investigated whether CNO and NDC can affect microglial survival in cultures prepared from Cx3CR1GFP/+, heterozygous mice, which showed green fluorescence in microglia. Our results showed that CNO and NDC alone or even in combination with LPS had no effect on GFP-positive microglia cell number in neuron-glia cultures up to 7 days after treatment (Additional file 1: Figure S1).

LPS-induced microglial activation (M1 phenotype) is associated with the release of proinflammatory factors. The release of the proinflammatory factors at their peak release times, including superoxide (15 min), TNF-α (3 h), and nitric oxide (NO, 24 h) [43–45], were subsequently detected in culture supernatants to assess the role of CNO and NDC on LPS-induced microglial activation. First, we showed that pretreatment with CNO or NDC reduced the release of extracellular superoxide in LPS-challenged neuron-glia cultures (Fig. 4a, b). Similar inhibitory effects of these two metabolites, but less potent, were observed for the release of TNF-α and NO (Fig. 4c–f). It is interesting to note that CNO- and NDC-elicited suppression of NO, TNF-α, and superoxide displayed a similar pattern, i.e., a bell-shaped dose response curve; the most effective inhibitory doses were 0.01 and 0.1 μM for CNO and NDC, respectively, which was identical to those in DA neuroprotection (Fig. 1). These results suggest that CNO and NDC may modulate inflammation-driven dopaminergic neurodegeneration by inhibiting microglial activation and subsequent release of these proinflammatory factors.

### Microglial NOX2 is essential in mediating the anti-inflammatory and neuroprotective effects of CNO and NDC

**Fig. 5:**
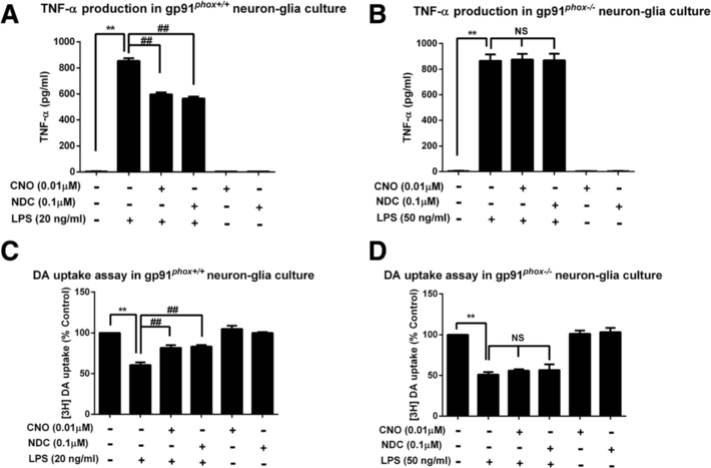
Gp91^*phox*^ genetic ablation blocks the anti-inflammatory and neuroprotective effects of CNO and NDC. Neuron-glia cultures prepared from wild-type (gp91^*phox+/+*^ *)* and NOX2-deficient (gp9 ^*phox−/−*)^ mice were pretreated with vehicle or CNO (0.01 μM) or NDC (0.1 μM) for 30 min followed by LPS (20 ng/ml for gp91^*phox+/+*^or 50 ng/ml for gp9^*phox−/−*^) stimulation. **a**, **b** The supernatant levels of TNF-α were detected 3 h after LPS treatment. **c**, **d** The protective effects of CNO and NDC against LPS-induced dopaminergic damage were assessed by DA uptake assay 7 days after treatment. Results are expressed as the mean ± SEM or as a percentage of vehicle controls (mean ± SEM) from three independent experiments performed in triplicate. **p < 0.01, ^##^ *p* < 0.01. *NS* not significant

Among the proinflammatory factors triggered by LPS, release of superoxide was the most severely inhibited by both CNO and NDC. Previous reports from our laboratory indicated that NOX2 is the major enzyme in microglia that produces extracellular superoxide anions and is a major contributor to the increase of the intracellular reactive oxygen species (ROS), which in turn enhances TNF-α production [46]. We further studied the role of microglial NOX2, the key superoxide-producing enzyme in mediating the neuroprotective effects of clozapine. For this purpose, cell cultures prepared from mutant mice deficient in gp91^*phox*^, the catalytic subunit of NOX2, and from wild-type (gp91^*phox+/+*^) mice were used. Because dopaminergic neurons cultured from gp91^*phox−/−*^ mice are more resistant than that from gp91^*phox+/+*^ mice to LPS-induced neurotoxicity [47, 48], different concentrations of LPS were used for both types of cultures in order to produce equivalent loss of dopaminergic neurons: 50 ng/ml for gp91^*phox−/−*^ culture and 20 ng/ml for gp91^*phox+/+*^ culture. The results showed that pretreatment with CNO or NDC significantly attenuated LPS-induced TNF-α release and decreased DA uptake capacity in gp91^*phox+/+*^ cultures (Fig. 5a, c). In contrast, in gp91^*phox−/−*^ culture, CNO and NDC failed to show any effect on LPS-induced production of TNF-α and reduction of [^3^H]DA uptake capacity (Fig. 5b, d). These results suggest that NOX2 plays an indispensable role in CNO and NDC-afforded neuroprotection.

**Fig. 6:**
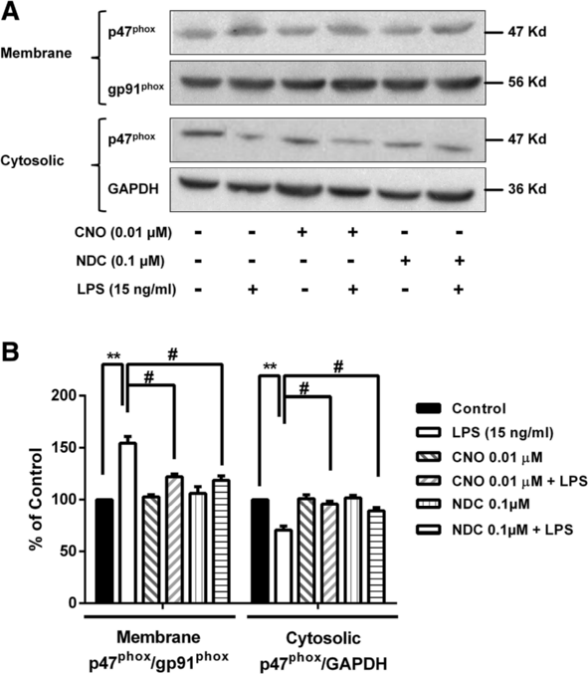
CNO and NDC inhibit NOX2 activation. **a** HAPI microglia cells were pretreated with CNO (0.01 μM)/NDC (0.1 μM) for 30 min followed by LPS stimulation. Fifteen minutes later, cells were harvested. The levels of p47^phox^ were determined in both membrane and cytosolic fractions using Western blot. GAPDH and gp91^phox^ were used for internal controls of cytosolic and membrane fractions, respectively. **b** The band density of blots were quantified. Results are expressed as a percentage of PMA group or vehicle controls (mean ± SEM) from three independent experiments performed in triplicate. **p < 0.01, ^#^ *p* < 0.05, ^##^ *p* < 0.01

To determine the mechanism of how CNO and NDC inhibit NOX2 activation, we determined the inhibitory effects of CNO and NDC on membrane translocation of cytosolic subunits of NOX2, an essential step for the activation of NOX2, in HAPI microglia cells. Western blot analysis showed that the levels of p47^*phox*^ was decreased in the cytosolic fraction but increased in membrane fractions in LPS-stimulated HAPI microglia cells (Fig. 6), indicating membrane translocation of p47^*phox*^. As shown in Fig. 6c, the changes of p47^*phox*^ in both cytosolic and membrane fractions induced by LPS were suppressed by CNO or NDC treatment. No significant change of p47^*phox*^ levels was observed in CNO or NDC alone-treated cells, compared with vehicle control. Quantitative analysis supported our observation by restoring p47^*phox*^ levels in both cytosolic (from 70.62 % of LPS alone back to 95.65 and 89.01 % by CNO and NDC, respectively) and membrane fractions (from 154.2 % of LPS alone back to 122.18 and 118.83 % by CNO and NDC, respectively, Fig. 6c). These results suggest that CNO and NDC inhibited NOX2 activation in microglia by interfering with the membrane translocation of NOX2 cytosolic subunits.

### CNO attenuates MPTP-induced dopaminergic neuron damage and motor deficits without showing granulocyte toxicity

**Fig. 7:**
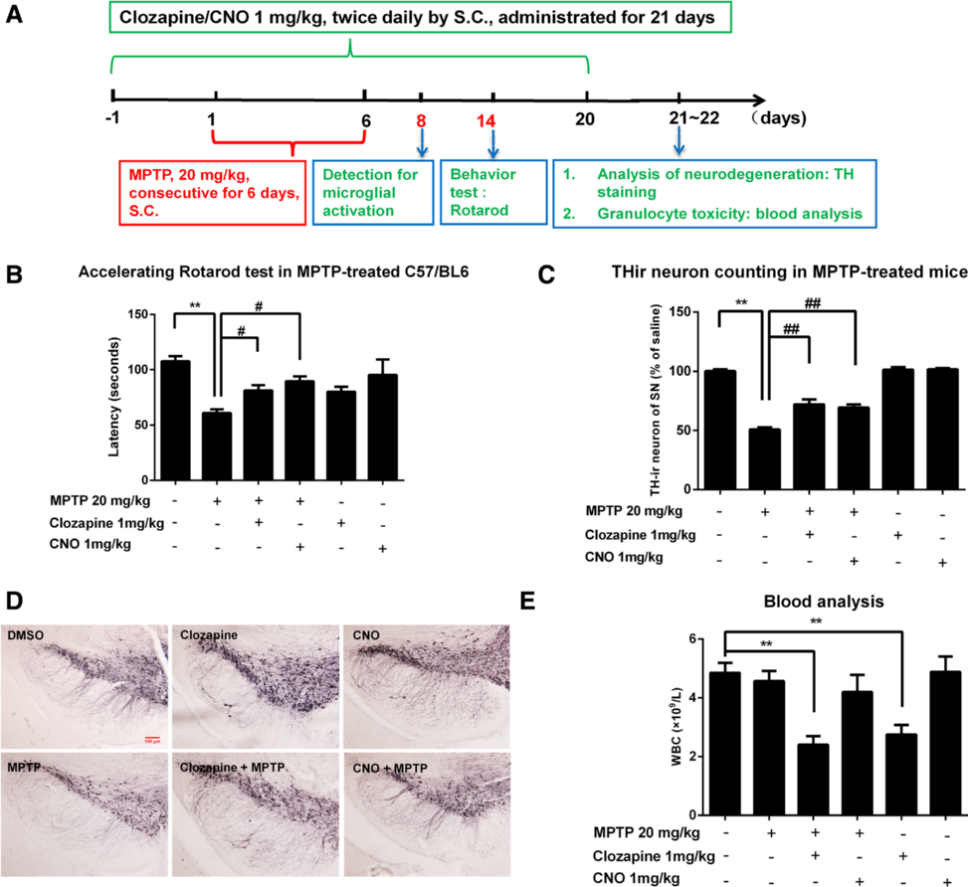
CNO and clozapine are equipotent in attenuating MPTP-induced dopaminergic neuron damage and motor deficits but show differential effects on blood WBC counts. **a** Experimental designs. Eight-week-old male C57BL/6J mice received daily MPTP injections (20 mg/kg, s.c.) for six consecutive days. From 1 day prior to MPTP injection, clozapine or CNO (1 mg/kg, s.c.) was administered twice daily for 21 consecutive days. **b** Fourteen days after initial MPTP injection, the protective effects of clozapine and CNO against MPTP-induced motor deficits were measured by the rotarod test. **c** Twenty-one days after the first injection of MPTP, dopaminergic neurons in the SNpc were immunostained with anti-TH antibody and the numbers of THir cells in SNpc were counted. **d** The representative images of TH staining in the SN were shown. **e** Blood WBC counts were shown in clozapine- and CNO-treated mice. Results are expressed as the mean ± SEM or as a percentage of vehicle controls (mean ± SEM). **p < 0.01, ^#^ *p* < 0.05, ^##^ *p* < 0.01; *n* = 4–8. *Scale bar* = 100 μm

To further verify the neuroprotective effects of clozapine metabolite in vivo, a MPTP mouse PD model was used. Since CNO and NDC are equally potent in protecting dopaminergic neurons against inflammation-induced damage, we selected CNO only to compare the dopaminergic neuroprotective effect with clozapine. One day prior to MPTP injection, clozapine or CNO (1 mg/kg, s.c.) was administered twice daily for 21 consecutive days. The protective effects of clozapine and CNO against MPTP-induced motor deficits in mice were measured using accelerating rotarod test at 14 days after initial MPTP injection (Fig. 7a). Consistent with our previous report [49], MPTP injection reduced the rotarod activity in mice by about 43 % of latency time on the accelerating rod. Both CNO and clozapine treatment significantly ameliorated MPTP-induced deficits of rotarod activity with equal-potency (Fig. 7b). In addition to ameliorating motor deficits, CNO and clozapine treatment displayed potent efficacy in attenuating MPTP-induced dopaminergic neurodegeneration (Fig. 7c). MPTP treatment caused 50 % loss of THir neurons in the SNpc, which were reduced to 28 % in mice pretreated with CNO and clozapine (Fig. 7c, d).

To detect the neutropenia of both clozapine and CNO, the number of WBC in whole blood samples prepared from mice treated with CNO and clozapine. Interestingly, unlike parent compound clozapine, CNO had no significant toxicity on neutrophils even combined with MPTP up to 21 consecutive days of treatment (Fig. 7e).

### CNO attenuates MPTP-induced reactive microgliosis

**Fig. 8:**
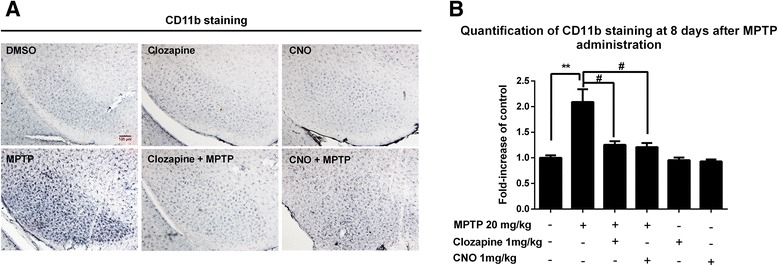
CNO and clozapine attenuate MPTP-induced reactive microgliosis with equi-potency. **a** Eight-week-old male C57BL/6J mice started to receive 1 mg/kg clozapine or CNO (s.c., twice daily) 1 day before MPTP injection (20 mg/kg, subcutaneously for six consecutive days). After 8 days of initial MPTP injection, microglia in the SN were stained with anti-CD11b antibody and the representative images were shown. **b** The inhibitory effects of clozapine or CNO on microglial activation were determined by analyzing the density of CD11b immunostaining. Results are expressed as a percentage of vehicle controls (mean ± SEM). **p < 0.01, ^#^ *p* < 0.05; *n* = 4–8. *Scale bar* = 100 μm

To determine whether CNO-afforded dopaminergic neuroprotection was linked to inhibition of microglial activation in vivo, we stained nigral microglia with CD11b, an alpha chain of the β2 integrin receptor, 8 days after MPTP injection. In MPTP-treated mice, activated microglia characterized by a hypertrophied morphology and intensified CD11b staining were observed throughout the nigral reticulate area (Fig. 8a). Analysis of CD11b density supported these morphological observations. Compared with the MPTP alone group, both clozapine and CNO treatment markedly attenuated microglial activation, as shown by a reduced density of CD11b staining (Fig. 8a, b).

## Discussion

In this study, we demonstrated that CNO and NDC, two major metabolites of clozapine, exert potent anti-inflammatory and neuroprotective effects in MPTP- and inflammation-generated dopaminergic neurotoxicity. CNO displayed the same potent efficacy as clozapine in protecting DA neurons in MPTP-mediated PD mouse model. Further, in vitro studies revealed a dose-related correlation of anti-inflammatory effect and their potency in neuroprotection by both CNO and NDC. Mechanistic studies indicated that inhibition of microglial NOX2-generated superoxide is the major target for the anti-inflammatory actions: a novel pharmacological property shared by both clozapine and its two metabolites. This study clearly demonstrates that CNO and NDC exhibit potent central effects and may contribute to some of the neuropsychopharmacological actions of clozapine.

Neuroinflammation mediated by microglia is known to be accompanied by oxidative stress, a common pathogenic pathway for neurodegenerative process and psychiatric syndromes [50, 51]. Either overproduction of oxidants such as ROS, or deficiencies in antioxidant defense, or some combination thereof, can perturb the redox homeostasis, resulting in oxidative damage [52]. Though in the process of neuroinflammation, both microglia and astrocytes play critical roles in counterbalancing the neurotoxicity or neuroprotective effect to a variety of cytotoxic insults [53]; results from this study and our previous report provided a novel anti-inflammatory mechanism of actions of clozapine and its metabolites as we found that microglia were essential for the neuroprotective effect of CNO or NDC (shown in Fig. 2, protective effect disappeared as microglia were depleted). Among the proinflammatory factors released by activated microglia, superoxide production was the most severely inhibited by CNO and NDC. Superoxide is one of the prominent factors released by activated microglia, and NOX2 has been identified as a major source [54]. In addition to extracellular ROS, NOX2 also contributes to increase intracellular ROS that is a crucial secondary messenger for microglial signaling and proinflammatory properties [46, 55]. We found that both CNO and NDC inhibited NOX2-derived superoxide production. Moreover, the anti-inflammatory and neuroprotective effects of CNO and NDC were abolished once the gene of *NOX2* was ablated, suggesting that NOX2 is the action target of CNO and NDC. Our results suggest that the inhibitory effects of CNO and NDC are independent of neurotransmitter receptors but directly through acting on NOX2. This possibility is consistent with the report showing that clozapine is able to bind to neutrophil NOX2 in vitro [56]. Binding experiments for CNO/NDC to microglial NOX2 should be guaranteed in our future studies.

Clozapine is an atypical antipsychotic medicine used to treat schizophrenia in patients whose symptoms are not controlled with standard antipsychotic treatment. Despite intensive studies on various G protein-coupled receptors (GPCR), such as monoaminergic receptors and muscarinic receptors [11, 12], mechanisms underlying the antipsychotic action of clozapine remain unclear. Recent advance in the research of neuroinflammation has provided a new avenue to uncover mechanisms underlying the pathogenesis of neurodegenerative diseases and mental disorders [57, 58]. There has been an increasing recognition of the pathogenic role of microglia-mediated neuroinflammation in psychiatric diseases such as depression, bipolar, schizophrenia, and obsessive-compulsive disorder [22–24]. For instance, both postmortem and PET studies have provided evidence for the existence of microglial activation in the brains of schizophrenia patients [59–61]. Some clinical trial studies have demonstrated a beneficial effect of adjunctive minocycline, a well-known anti-inflammatory compound, on the negative symptoms of schizophrenia [62, 63] and on cognitive function [63]. Indeed, a wide variety of psychoactive drugs, such as antidepressants and antipsychotics, have been shown to suppress microglial activation [23, 47, 64, 65].

Earlier report from our laboratory illustrating the anti-inflammatory and neuroprotective action of clozapine lends further support to the notion that neuroinflammation may play a role in schizophrenia [25]. Current study further extends our previous report indicating that CNO and NDC, which are not shown to be centrally active metabolites, possess both anti-inflammatory and neuroprotective actions with equal or even higher potency than that of clozapine. These findings raise a possibility that these two metabolites may be used as anti-inflammatory and neuroprotective agents for treating neurodegenerative diseases or even as adjunctive drugs for treatment of various mental diseases. Given that a relatively high risk of neutropenia or agranulocytosis greatly hampers the long-term usage of clozapine, if future studies find CNO or NDC effective clinically in treating certain CNS diseases, these two metabolites would possess a great advantage over clozapine in terms of potential toxicities. It has been reported that NDC is much less toxic to neutrophils in comparison to clozapine and CNO shows no toxicity at all at concentrations up to 100 μM [8, 66]. For this reason, exploring the potential therapeutic use of CNO may be of great interest for two reasons: (1) CNO shows higher potency in both anti-inflammatory and neuroprotective effects than that of NDC and clozapine in LPS-treated neuron-glial cultures; (2) Consistent with previous reported in vitro study, our study showed that CNO had no significant toxicity on neutrophils in vivo even combined with MPTP for up to 21 consecutive days of treatment. Currently, testing the antipsychotic efficacy of CNO using schizophrenia animal models is underway in our laboratory to determine the possibility that CNO can be effective as a substitute of clozapine in treating this mental disease.

On the other hand, currently, the role of astrocyte in PD remains controversial. We recently demonstrated that in LPS-treated neuron-glia cultures, astrocytes tend to protect DA neurons against neuroinflammation-mediated degeneration through secretion of neurotrophic factor GDNF [53]. We further revealed that astroglia may not possess the capability to directly response to the innate immune stimuli LPS, but rather depend on crosstalk with microglia. However, the protective effects of astrocyte against MPP^+^-induced DA degeneration in vitro were not observed in our previous study, although the reason still remains unclear [67]. Saijo et al. [68] reported that astrocyte is not only non-neuroprotective, but even amplified the proinflammatory response in microglia, resulting in exacerbated DA degeneration. Despite of these conflicting results, targeting astrocyte, such as cystine/glutamate exchange transporter [69], glutathione synthesis, [70] and 5-HT(1A) receptor [71], exhibited potent neuroprotective effects in multiple models of PD. Therefore, future studies focusing on the role astrocyte in the neuroprotective effects elicited by CNO and NDC should be guaranteed, which may provide new opportunities for developing novel strategy for PD therapy.

## Conclusions

In summary, our study shows that NDC and CNO, two major metabolites of clozapine, share the same anti-inflammatory and neuroprotective effect with their parent drug. This study suggests that metabolites of clozapine may also contribute to the therapeutic effect of the drug in the treatment of schizophrenia with the mechanism independent of neurotransmitter receptors. It also warrants animal study to investigate the in vivo metabolism and antipsychotic effect of NDC and CNO to evaluate the potential of NDC and CNO as substitute drugs for clinical use as they are less toxic to neutrophils.

## Additional file

Additional file 1: Figure S1. CNO and NDC had no effect on the number of microglia. Mesencephalic neuron-glia cultures were prepared from Cx3cr1gfp/+, heterozygous mice. Cultures were pretreated with vehicle or indicated concentrations of CNO or NDC for 30 min before the addition of LPS (15 ng/ml). After 7 days of treatment, no significant difference of the number of microglia (green) in each group was detected. Representative images of microglia at 7 days after treatment in each group were shown. Scale bar = 50 μm. (DOC 180 kb)

## Abbreviations

AD: Alzheimer’s disease
ANOVA: analysis of variance
CNO: clozapine *N*-oxide
CNS: central nervous system
DA: dopamine
GPCR: G protein-coupled receptors
Iba-1: ionized calcium-binding adaptor molecule 1
LME: Leu methyl ester
LPS: lipopolysaccharide
MPTP: 1-methyl-4-phenyl-1,2,3,6-tetrahydropyridine
NDC: *N*-Desmethylclozapine
NO: nitric oxide
NOX2: NADPH oxidase
PD: Parkinson’s disease
ROS: reactive oxygen species
SCZ: schizophrenia
SEM: standard error of mean
SN: substantia nigra
SNpc: SN pars compacta
TH: tyrosine hydroxylase
THir: TH-immunoreactivity
TNF-α: tumor necrosis factor-α
