# Supplementary material for: Clozapine metabolites protect dopaminergic neurons through inhibition of microglial NADPH oxidase
Source: J Neuroinflammation. 2016 May 16;13:110. doi: 10.1186/s12974-016-0573-z (PMC4869380; doi:10.1186/s12974-016-0573-z)
Supplement: Additional file 1: Figure S1. — CNO and NDC had no effect on the number of microglia. Mesencephalic neuron-glia cultures were prepared from Cx3cr1gfp/+, heterozygous mice. Cultures were pretreated with vehicle or indicated concentrations of CNO or NDC for 30 min before the addition of LPS (15 ng/ml). After 7 days of treatment, no significant difference of the number of microglia (green) in each group was detected. Representative images of microglia at 7 days after treatment in each group were shown. Scale bar = 50 μm. (DOC 180 kb) [file 12974_2016_573_MOESM1_ESM.doc]

**
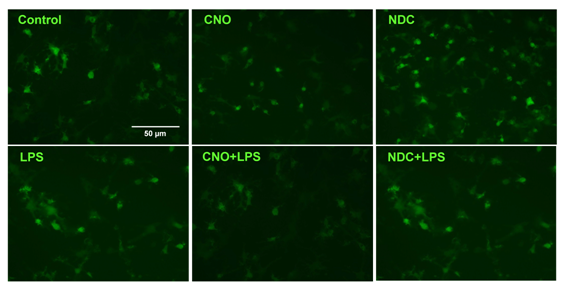
**

**Additional file 1: Figure S1. CNO and NDC had no effect on the number of microglia.** Mesencephalic neuron-glia cultures were prepared from Cx3cr1gfp/+, heterozygous mice. Cultures were pretreated with vehicle or indicated concentrations of CNO or NDC for 30 min before the addition of LPS (15 ng/ml). After 7 days of treatment, no significant difference of the number of microglia (green) in each group was detected. Representative images of microglia at 7 days after treatment in each group were shown. Scale bar = 50 µm.
